# “Some They Need Male, Some They Need Female”: A Gendered Approach for Breast Cancer Detection in Uganda

**DOI:** 10.3389/fgwh.2022.746498

**Published:** 2022-03-25

**Authors:** Deborah Ikhile, Damilola Omodara, Sarah Seymour-Smith, David Musoke, Linda Gibson

**Affiliations:** ^1^Department of Primary Care and Public Health, Brighton and Sussex Medical School, University of Sussex, Brighton, United Kingdom; ^2^Global Public Health, Institute of Population Health Science, Barts and The London School of Medicine and Dentistry, London, United Kingdom; ^3^Department of Psychology, School of Social Sciences, Nottingham Trent University, Nottingham, United Kingdom; ^4^School of Public Health, Makerere University, Kampala, Uganda; ^5^Public Health, Institute of Health and Allied Professions, School of Social Sciences, Nottingham Trent University, Nottingham, United Kingdom

**Keywords:** breast cancer, gender, gender disparities, Uganda, intersectionality, gendered approach

## Abstract

**Introduction:**

There are several challenges associated with breast cancer detection in Uganda and other low-and-middle-income countries. One of the identified challenges is attributed to the health workers' gender, which facilitates gender disparities in access to breast cancer detection services. Although this challenge is well acknowledged in existing literature, there are hardly any studies on how it can be addressed. Therefore, drawing on an intersectionality lens, our study examined how to address gender disparities facilitated by health workers' gender in accessing breast cancer detection services in Uganda.

**Materials and Methods:**

We collected qualitative data through semi-structured interviews with twenty participants comprising community health workers, primary health care practitioners, non-governmental organizations, district health team, and the Ministry of Health. For the data analysis, thematic analysis was conducted on NVivo using Braun and Clarke's non-linear 6-step process to identify the themes presented in the results section.

**Results:**

Four themes emerged from the data analysis: understanding a woman's gender constructions; health workers' approachability; focus on professionalism, not sex; and change in organizational culture. These themes revealed participants' perceptions regarding how to address gender disparities relating to the role health workers' gender play in breast cancer detection. Through the intersectionality lens, our findings showed how gender intersects with other social stratifiers such as religious beliefs, familial control, health worker's approachability, and professionalism within the health workforce.

**Conclusion:**

Our findings show that the solutions to address gender disparities in breast cancer detection are individually and socially constructed. As such, we recommend a gendered approach to understand and redress the underlying power relations perpetuating such constructions. We conclude that taking a gendered approach will ensure that breast cancer detection programs are context-appropriate, cognizant of the prevailing cultural norms, and do not restrict women's access to breast cancer detection services.

## Introduction

Breast cancer is a major cause of premature deaths in low- and middle-income countries (LMICs), where women bear the greatest burden of the disease (1). It is estimated that one in five women in Africa dies of the disease (1). Previously associated with high-income countries (HICs), breast cancer now disproportionately causes premature deaths among women in LMICs (2, 3). The increasing burden of breast cancer in LMICs is associated with epidemiological transition, demographic patterns, urbanization, westernization (4). There exists disparity in breast cancer incidence and mortality across different regions. For instance, the estimated incidence of breast cancer in Europe is 531,086, almost three times the incidence in Africa 186,598 in 2020 (5). Even though there has been a consistent increase in breast cancer incidence in Africa, the current estimate still seems low (4). This low incidence has been attributed to inadequate research, poor breast cancer monitoring and insufficient cancer registries in this region (4). In relation to mortality, current estimates show that ~ 46% of people with the disease in Africa die from it, compared to 27% in Europe (5).

In Uganda, breast cancer has tripled over the past three decades (6, 7) and currently accounts for the second most common cancer among women, with an estimated mortality rate of 51.8% (5). This high mortality rate is primarily attributed to late detection, late diagnosis, and inadequate access to follow-on treatment facilities in Uganda (8). Recent studies have showed that between 80 to89% of breast cancer in Uganda are detected at stages III and IV (8, 9). Improving earlier breast cancer diagnosis, ideally, at stages I and II requires a commitment to early detection strategies in addition to prompt access to treatment services (10).

The recommended evidence-based early breast cancer detection strategies for Uganda where cancer resources are constrained include culturally sensitive and linguistically appropriate breast education, Breast self-examination (BSE), and Clinical Breast Examination (CBE) (10, 11). Although BSE and CBE have been discredited in HICs such as the USA due to insufficient scientific evidence to support their efficacy (12), both strategies are still recommended as feasible, cost-effective, and appropriate for breast cancer detection in LMICs like Uganda (10, 13). The availability and provision of these breast cancer detection strategies in Uganda varies across the health care system. Uganda has a decentralized health care system whereby health services are delivered within seven tiers, including national referral hospitals, regional hospitals, district hospitals, health center IV, health center III, health center II and community health workers (CHWs), locally referred to as the Village Health Teams (VHTs) (14, 15). Two recent situational analyses of the breast cancer detection services in the country (8) and specifically within the PHC system (health centers I to IV) (16) both reveal that breast cancer detection services are currently lacking in health centers I and II. In health centers III and IV, breast education and BSE are occasionally promoted, and CBE is sometimes performed only for symptomatic women.

There are several challenges associated with breast cancer detection in Uganda, including low awareness of breast cancer risk factors and symptoms among women (17, 18); low knowledge and practice of BSE among women; low awareness of breast cancer symptoms and detection strategies among health workers (8); lack of national breast cancer detection guidelines (19); geographical barriers (20); and sociocultural factors (19, 21). This current study focused on the sociocultural factors, specifically the prevailing sociocultural norms around the health worker's sex and the provision of breast cancer detection services in Uganda. The terms “sex” and “gender” are used to designate the interrelationship among biological and sociocultural identities. Sex constitutes the physical characteristics that are biological and physiological, which differentiate males from females (22). Gender, on the other hand, is defined as a socially constructed identity and roles that societies ascribe to the sexes that revolve around issues of femininity and masculinity (22). We refer to health workers' gender in this paper because the challenge is not posed by the biological identity of the health workers but rather the social and cultural characteristics ascribed to their sexes. The prevailing sociocultural norm in Uganda perpetuates gender disparities around access to health service delivery, as women would not feel comfortable exposing their breasts to a male health worker to palpate. A study conducted among women in the central region of Uganda confirmed that one of the barriers to the early detection of breast cancer is that it is culturally inappropriate for a woman to openly discuss or allow a man to examine her breasts (19). These perceptions are not only shared by women themselves. In a recent survey with primary health care providers in Uganda, most of them also perceived that male health workers would not be able to provide breast cancer detection services (16). This sociocultural norm around male health workers providing health services have also been linked to cervical cancer screening in sub-Saharan Africa (23). The issue of health worker's sex in breast cancer detection is problematic in a country where the health workforce is dominated by males (24).

The health worker's gender does not operate as an isolated challenge to early breast cancer detection, as this could intersect with other social factors to produce different decision-making experiences among women. In Uganda, the context of the current study, gender disparity in late breast cancer detection can be further compounded by intersecting identities and a range of social factors such as geographical location, religions, and socioeconomic status (SES) of the women (16). Besides, it is important to acknowledge that although these social categories are often considered separately, they are intersectional and interrelated (25, 26). Moreover, multiple dimensions and overlapping identities make up a whole person to help understand their lived experience. A number of theoretical frameworks illuminate the diversity of experiences among women in accessing healthcare services (27). One of such is what has become recently known as intersectionality in health services research (28, 29). The intersectionality framework originated from the work of African American feminist researchers studying women and social identity (25, 26). An intersectionality framework recognizes sex and gender are not uniform or discrete and are therefore not easily separated (30). The framework further enhances understanding of how “aspects of social status (e.g., gender, race, socioeconomic status, and sexuality) are understood to affect health outcomes in complex, multiplicative ways that can never properly be captured by attempts to parcel out the individual contributions of single social domains” (30). Understanding how gender interacts with other social stratifiers like SES can provide new knowledge on how to address gender disparities effectively (31). Therefore, we adopted an intersectionality lens to understand the multiple social stratifiers that intersect with gender to produce disparities in access to breast cancer detection services among women in Uganda.

Although gender is a crucial determinant of health (31), it is still underexplored in Uganda and other LMICs (32, 33). Specifically, there are hardly any studies on the influence of health workers' gender and gender disparities in breast cancer detection in Uganda. Existing studies have focused on addressing the challenges of breast cancer detection in Uganda, mainly providing recommendations on how to address individual and health system challenges. To date, no known study has explored how the challenge of gender disparities in breast cancer detection in Uganda can be addressed. Therefore, using the intersectional lens, this current study examined how to address gender disparities facilitated by health workers' gender in accessing breast cancer detection services in Uganda. Specifically, it provided context-appropriate evidence required to ensure future breast cancer detection interventions are grounded in the sociocultural realities of the women.

## Materials and Methods

### Study Setting

This study was conducted in Kajjansi Town Council, a peri-urban community in Wakiso district, the central region of Uganda. Uganda has an estimated population of 45.7. million, where 50.7% of these are composed of females (34). Wakiso district is engulfed within Kampala, the capital city, and is the most populous district in the country, with an estimated population of 2.7 million (35). The study setting, Kajjansi town council was purposively selected for this study because previous research on the challenges to early detection of breast cancer had been conducted in the area by DI where women identified the health worker's sex as a challenge (19). Access to health services in the town council is through public and private health facilities. Public health facilities in the Town Council are within the PHC system and organized as health center IV, health center III, health center II and the services provided by CHWs (16). Although the Government supports the public PHC system, it is faced with numerous challenges, including the inadequate provision of comprehensive health services, health workforce shortages, drug stock out (16, 36).

### Study Design

The findings presented in this paper are part of a doctoral research project that examined how Uganda's primary health care system can be strengthened to promote early breast cancer detection (16). The doctoral research used an explanatory sequential methodology, whereby qualitative data collection built on findings from an initial quantitative phase. Qualitative research was considered useful for providing detailed and emergent findings (37, 38). The findings presented in this paper draw on the qualitative phase. Our paper focuses on how the challenge of health workers' gender impedes access to breast cancer detection services for women in Uganda. We employed a thematic analysis approach to analyze the semi-structured interviews with healthcare workers about their insights on how to address this challenge. The use of semi-structured interviews enabled the data to be collected in a conversational style while guided by a set of open-ended questions (39). The open-ended questions and prompts were informed by findings from the quantitative phase of DI's doctoral research (now completed). The qualitative phase of this study was conducted virtually using Skype. Although interviews were traditionally conducted via face-to-face interactions, virtual platforms have become widely recognized and invaluable for contemporary research to mitigate different constraints to accessing study participants physically (40, 41). Further information on the virtual interviews for this study have been detailed in a different publication co-authored by DI (40). The interview questions were piloted via Skype to test the appropriateness of the questions and the virtual platform.

### Data Collection Process

**Figure 1:**
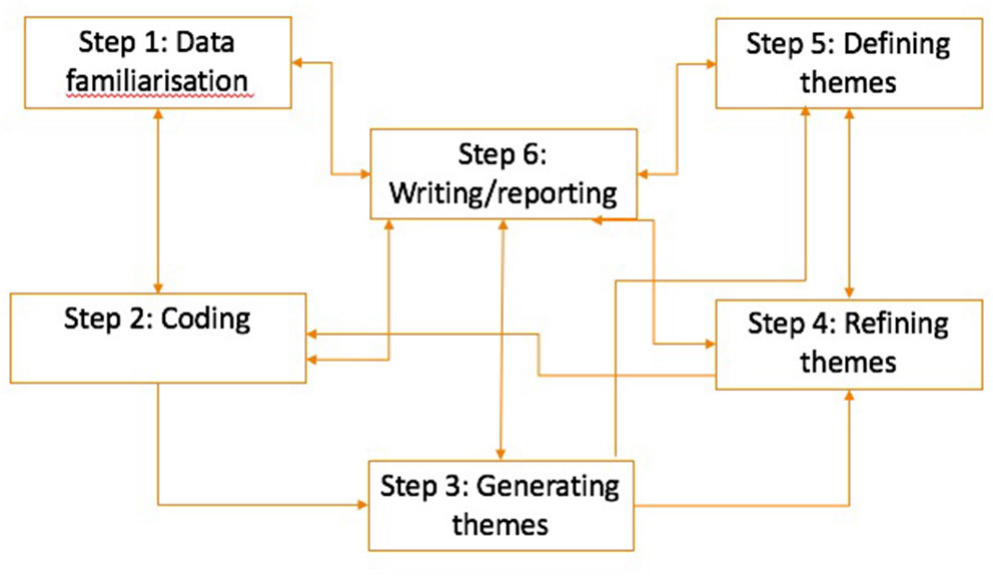
Thematic analysis process.

The qualitative data was collected from January to June 2019 through virtual semi-structured interviews using Skype. Twenty-five semi-structured interviews were conducted among 14 CHWs, three (3) district and national health officials, three (3) primary health care practitioners (PHCPs), and five (5) representatives from non-governmental organizations (NGOs) (Figure 1). The PHCPs were professional health workers like clinical officers, midwives, and nurses in health centers II, III, and IV. Our study conceptualized PHC workers as PHCP and CHWs. The data was collected until data saturation was achieved.

Access to the study site and participants was facilitated through a long-term research partnership between Nottingham Trent University, in the UK, and Makerere University in Uganda (42). DI also had established access to the study site and relationships with the CHWs and PHC workers as the data presented in this paper were collected as part of her doctoral research project. Also, DM who is Ugandan supported with recruitment of participants from NGOs, as well a district and national health stakeholders. The participants were recruited through purposive sampling and snowballing, and each interview was audio-recorded using an external recording device. The participants were purposively selected for their first-hand experience of engaging with women in primary health care delivery and their ability to converse in English. The ability of the participants to speak English was an important criterion to avoid the need for a translator which would have been difficult via Skype. The interviews were conducted by DI, a Black African researcher and lasted between 30 to 90mins. The interviews for the CHWs were conducted in a field office located in the study site, while the others were conducted at their work place. All the interviews were conducted with the participants alone in the room.

### Data Analysis

**Figure 2:**
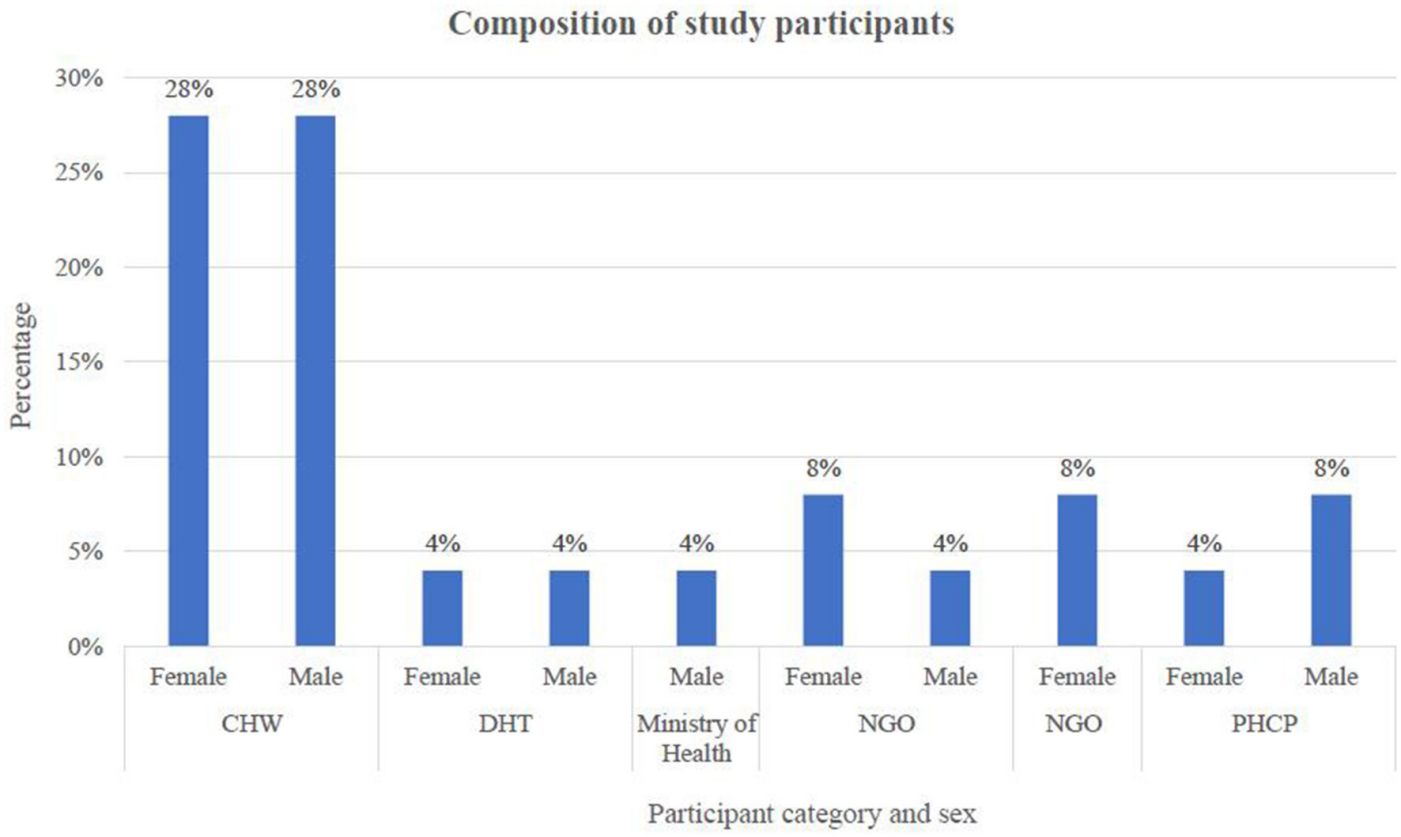
Composition of study participants involved in the interviews.

For the data analysis, an inductive thematic analysis, guided by Braun and Clarke's (2006) iterative 6-step thematic analysis process was followed (Figure 2) to identify the themes presented in the results section (43).

The first step, data familiarization, involved verbatim transcription of the audio-recorded interviews and preliminary identification of codes by DI. The next step was the coding process which involved disassembling the data into meaningful categories. The coding for this study was data-driven and done using QSR NVivo 12, a qualitative data analysis software. DI developed the initial codes as part of her doctoral research which were reviewed and revised by LG, SSM and DM. After coding, DI generated the themes through the categorization of the codes. Steps four and five involved refining and defining the themes, respectively. The themes generated in step three were refined by DI and DO by categorizing them into overarching themes and sub-themes. Afterward, the themes were renamed where required and then described by DI and DO. The last step was writing up the analyzed data in a report format and supporting these with verbatim extracts from the transcripts. We adopted a combination of illustrative and analytical approaches for writing up the analyzed data (44).

### Ethics Approval

The study obtained ethics approval from the Nottingham Trent University College of Business, Law and Social Sciences Research Ethics Committee (2018/233). Since the interviews were conducted virtually, verbal consents were provided and recorded at the start of the interviews. The purpose of the research was explained to all participants and they were aware that the interview was being recorded. Participation in the study was made entirely voluntary for the participants, and participants were advised of their right to withdraw their participation or contribution to the study data. Names and personal information that would make it possible to identify participants were not included in the transcripts; an identification number was assigned to each participant. The anonymized transcripts were stored in a personal folder on Nottingham Trent University OneDrive managed by DI.

## Results

The following four (4) themes were identified from the data analysis: understanding a woman's gender constructions; health workers' approachability; focus on professionalism, not sex; and change in organizational culture. These themes revealed participants' perceptions regarding how to address gender disparities relating to the role health workers' gender play in breast cancer detection.

### Understanding a Woman's Gender Constructions

The first theme from this current study indicated that the perceptions around the influence of health workers' gender on accessing breast cancer detection services are based on individual preferences, which varied from one woman to another. The participants generally revealed that a woman's preference and her gender constructions are influenced by her experience, knowledge, beliefs, and marital status.

*Maybe you can go when you have already checked yourself, tell the doctor what you have felt, and ask for a female doctor to help you out if you are not comfortable with the male. Like Muslims [women], they are not allowed to be touched by men, so you can ask for what's okay with you (female CHW)*.

*I think it is a challenge...And by the way, even their husbands will not allow them to go to the health facilities if they will be seen by a male nurse there. So, I think that's also a challenge, but through health talks, it can help us. I think the health talks may not target women alone, even the men; for them, they are ignorant. I think we can also target men. (female PHCP)*.

In the first extract above, the female CHW reported how some women might not want their breasts checked by a male health worker because of their religious beliefs about being touched by a man who is not their husband. However, the first extract also suggested that attending health facilities for breast cancer detection is prompted by prior identification of symptoms by the woman during BSE. In the second extract, the female practitioner explained how the woman's preference is driven by her husband's authority. Even though both extracts orient to a choice/preference, these are shaped by the woman's sociocultural context, like her religious beliefs and husband's authority as the head of the household. According to the female practitioner, an understanding of these gender constructions can be achieved through sensitization (what the participant referred to as *health talks)* of the health workers, women, and men.

### Health Worker's Approachability

The individual preference presented in the theme above did not necessarily mean women would prefer to receive breast cancer detection services from female health workers. According to most of the participants, women were generally more comfortable with a health worker irrespective of their sex when they were polite, friendly, and confidential, thereby enabling women to discuss concerns with their breasts comfortably. However, most participants linked these polite, friendly, and confidential attributes with the male health workers. These views were shared by both male and female participants, as indicated from the quotes below:

*But most times our female health workers, you can find out that they are not approachable. Since they are not approachable, the client can be there with the pain and not want to go to her [female health worker]. So, our health workers need to be approachable. At times the males are very approachable; for them, they are very easy...Like someone has come to ask you any question, for those ladies, at times they don't want to give their time to talk to them or welcome them. At times those ladies [health service user] they need time because that breast, they need time to say I have this. If you don't give her time, she cannot tell you. That's why it depends on the health worker for them to be open. But for these male health workers, at times, they give them time- what's the problem? What can I do for you? They have that welcoming approach (male CHW)*.

*Sometimes, some they need male, some they need female. It depends on the way of approach and how someone talks to the people. Some of them need female and some of them they need male and sometimes, in our villages they need male because they know that they can't tell their problems...Sometimes these males, their approach is good, and sometimes, the way they handle the women is not the way women handle the women...I don't know whether women to women they are rude sometimes (female CHW)*.

In the extracts above, male health workers were constructed as giving good health care, approachable, spend time asking questions, polite (see second extract discussion of rude female health workers), and trustworthy. Also, the second extract by the female CHW shows how individual preference intersects with their social relations with male and female health workers.

### Focus on Professionalism, Not Sex

Most of the participants were of the opinion that the gender disparities as a result of the perceptions around health workers' gender can be addressed by focusing on the PHCP's profession as a doctor, nurse, or midwife. The participants perceptions suggest that the health workers' gender should be shaped by professional identity rather than their gender identity.

*Hmmm, things regarding life there is no need saying I want a female or male since they are going to give you the service in a good condition. You have to let him or her do the service because you've come for the service. So, we should not segregate that male or female. All health workers, regardless of their sex, are there to provide health services to their clients (female CHW)*.

*For us VHTs, we work together male and female unless in the hospital when you have a problem, a doctor is a doctor even if he is a male, you have to go for medical check-up (female CHW)*.

The two extracts above both work up this notion of professional role being the dominant priority over gender constructions. This theme contrasts with the construction of choice and approachability presented in the first and second themes. This is because in the two extracts presented above, a woman's ability to indicate her preference, whether based on her gender constructions or the health worker's approachability, is undermined by the professional status of the health worker.

### Change in Organizational Culture

According to the study participants, the health workers' sex, either male or female, should not be a problem in an ideal PHC delivery system as women would be provided with an option to choose between female and male health workers. However, participants clarified that although the ideal requirement is to have the option offered to women that this is not usually the case in reality.

*You come to the facility, and whoever you find there is the one going to work on you. So, somehow, somewhere they just have to accept it even if they feel it is not comfortable...In that case, what is supposed to happen is normally when the health worker is a male, that male is supposed to be with a female nurse at least to be there by their side, and that is what is supposed to be done. Only that being in Africa somehow somewhere that does not happen. You find yes that the clinician is a male, and the woman comes, and this person is the one going to examine her. Also, there is need to inform the health workers about that; you know what, if you are male, then some women may not feel comfortable to examine, but you know because they study it in school and stop there. Like somewhere they forget about it, so they also need to remind them that much as you are a male clinician if at all a sensitive issue comes up, maybe like breast checking, at least you can work with female nurse so that the person is not feeling uncomfortable (female DHT)*.

*Seriously you know you can't serve all the people at their [individual] level, no. But that what I can say at least is to see that each health center or each department plus the hospital or whatever it has like two doctors- a male and the female. If someone fears the male, she can go to the female (female PHCP)*.

In the first extract above, the DHT representative indicated how agency is initially taken away from service users, “whoever you find there is the one going to work on you. So, somehow, somewhere they just have to accept it”. However, they also indicated that what should happen is that a female nurse would accompany a male doctor. The second extract also alludes to the restriction “you can't serve all the people at their level” but reinvokes the potential for choice if a male doctor is feared. Although these two extracts showed that the agency of choice outlined in theme one is not as simple, they also indicated that this could be made possible through a change in organizational culture that ensures the availability of options for women.

## Discussion

The current study examined how to address gender disparities as a result of the health worker's gender in breast cancer detection services in Uganda. Just as gender is socially constructed, the findings from our study were also socially constructed in that they were fraught with contrasting and subjective notions. Although the challenge of health worker's sex in breast cancer detection has been previously identified among Ugandan women (19, 21), this is the first known study to provide context-specific solutions on how this challenge can be addressed. It is important to note that the solutions provided were from the perspectives of PHC workers and other stakeholders with experience of engaging with women in PHC delivery. The challenge of health worker's sex is not unique to Uganda as it has been identified in other sub-Saharan African (SSA) countries such as Kenya (45) and Ghana (46). Still, there are no known studies in Uganda that have provided solutions to this challenge. Studies from other countries that have focused on addressing gender disparities have done so solely by addressing gender differences in breast cancer incidence between males and females (47). Therefore, despite the context specificity of our study, our findings provide novel approaches and considerations to address gender disparities relating to how the health worker's sex inhibits decision-making experiences and access to breast cancer detection services among women in Uganda.

Contrary to the perceptions from women as revealed through past studies that women generally preferred female health workers to examine their breasts (19, 48), findings from our study indicated that this may not always be the case, as such perceptions would vary from one individual to another. Therefore, our study findings indicated the need to be cognizant of and perhaps respect the factors shaping a woman's gender constructions and preference. One of the factors identified in this current study is religious beliefs. The influence of religious beliefs on health-seeking behaviors, specifically around breast cancer detection has been previously established (49, 50). A study in Ghana showed that Muslim women were less likely to perform BSE or undergo CBE than their Christian counterparts (49). Therefore, the collection of demographic data such as religion should not be for statistical or health resource planning purposes only but should inform the approach to healthcare delivery. Another factor identified from our study was the marital status of the woman. Existing literature on how marital status influences women's participation in breast cancer detection programmes show how this can either be an impediment (46, 51) or an opportunity for early detection (52, 53). While both perspectives were highlighted from our study findings, our findings showed that educating men can provide an opportunity to address gender disparities in access to breast cancer detection services.

Our study findings also highlighted that while understanding a woman's preference is important, the health worker's approachability can address disparities associated with their gender. This resonates with a past study in the UK which highlighted approachability as the most important factor influencing a women's preference for a particular gender (54). Approachability is one of the five dimensions of the patient-centered access to healthcare framework, with the other four being acceptability, affordability, availability and accommodation, and appropriateness (55). Approachability from our study findings was defined to embed confidentiality, politeness, trust, and friendliness. One of the significances of CHWs to health systems in resource-constrained settings is their proximity to the communities in which they serve, which gives them an insider positionality (56, 57). This can be both advantageous and disadvantageous. For instance, the insider positionality of CHWs has been recognized in previous literature as an asset to improve access to breast cancer detection programs (58, 59). However, our study findings revealed that such positionality could be disadvantageous, as this proximity could breed a lack of trust in female CHWs. Although CHWs in Uganda do not currently provide breast cancer detection services, it is important that these discourses around gender differences, approachability and positionality are taken into consideration when designing a breast cancer detection program to ensure optimum acceptability and utilization. Although our study findings further suggest that the gender differences around approachability apply to both PHCP and CHWs, this may not be the case as PHCP have a higher level of training (60), thus would exhibit a greater degree of confidentiality and professionalism. This point is supported by some participants in our study who opined that the health workers' gender should not be an issue, and the focus should rather be on professionalism.

Indeed, our third study finding considered the professionalism equated to PHC practitioners as a way of addressing gender disparities in accessing breast cancer detection services. Our study finding suggested that focus on the gender identity of PHCPs breeds disparities, whereas shifting the focus to professional identity would address such disparities. Such professionalism does not extend toward the CHWs. Although CHWs are part of the health care system, their voluntary status and recruitment via selection rather than undergoing a period of clinical training make them to be regarded as non-professional PHC workers. Since professionalism is equated to PHCPs as a result of their clinical training and qualifications, we surmise that when CHWs are upskilled through training and qualified to provide breast cancer detection services, their professional authority in that regard can be trusted. For example, a pilot study conducted in a rural community in Sudan that trained volunteer female health workers revealed that improving their knowledge and skills promoted the detection of breast cancer in asymptomatic women (50). Therefore, our stance that the health workers' gender may not be an issue when CHWs are trained and qualified to provide breast cancer detection services agrees with the study in Sudan. Perhaps, providing a breast cancer training composed of the technique of breast cancer detection and competencies in client interactions and understanding of gender constructions may even make PHCPs more approachable.

Although the focus on professionalism is a key perspective, this could undermine a woman's preference for a particular gender as it aligns with the dominance of the biomedical profession. Ascribed professionalism creates biases in services users (24), which inadvertently influences their preference. On the other hand, professionalism can also perpetuate the dominance of one healthcare profession over others which then disregards patients' voices as these healthcare professionals are regarded as experts. This dominance exerts an air of superiority which makes them distant from the everyday reality of women's lives, thus making them less approachable from women than non-professional PHC workers. Therefore, there is a need for further studies to explore the interaction between PHC workers' professionalism and approachability. The concept of professionalism may be more complex in SSA settings and vary across health issues. An example is in relation to maternal health, where women are attended to by a male or female doctor, and this does not restrict their access to care. In fact, studies in Uganda and other SSA countries have recorded high acceptance and preference for female PHC workers in relation to maternal and reproductive health issues (33). However, in the case of breast cancer detection or breast cancer care in general, women's health-seeking behavior is not based on their gendered role as mothers but rather on their individual identity as women. This suggests that there might be differences in how the health worker's gender is socially constructed across health issues. For instance, a 3-year study conducted among PHC workers in the UK revealed that the health worker's gender is an important consideration for women-specific issues. Thus, gender should not be taken as a generic or fixed construct, and its implication should be considered in relation to the specific health issue.

Our study further identified the need for a shift in the current organizational culture to address the disparities relating to health workers' gender. This shift requires a change in the PHC set up and practice from a restrictive system to an ideal PHC system. Firstly, an ideal PHC system should provide options in terms of skilled health workers to enable women to exercise their preference/choice for a male or female health worker. Implementing this option may be problematic for a setting like Uganda, which suffers from a high shortage of PHC workers (16) and even fewer females occupying higher cadres of the healthcare system (24). In Uganda, female health workers typically occupy a lower cadre like nursing, midwifery, and community health workforce (24). This is problematic for breast cancer detection as doctors and other higher cadres have more exposure to comprehensive health training, including breast cancer detection than lower cadre staff. Therefore, due to constraints of the health workforce in most SSA settings, it might be difficult, if not impossible, to request sex preference for a health worker. Where a male provides breast cancer detection services, we recommend the use of female chaperones e.g., female relatives or other female health workers like nursing assistants as a feasible and practicable option in a resource-constrained setting where traditional, cultural and religious barriers hinder access to breast cancer care.

The use of an intersectionality framework enabled a further understanding of the complex interaction between gender, individual beliefs, social relations, and professionalism within the health workforce. Choice for a particular gender came out strongly in our findings. But overall, a woman's choice from the perspectives of the study participants is influenced by cultural beliefs, husband's authority over her, approachability of health worker, and the PHC culture. Each of these intersecting factors can further facilitate or address disparities. A woman's gender constructions are affected by her cultural and religious beliefs, which prohibit her from receiving breast cancer detection services from the opposite sex. A further intersection observed from our study findings relates to familial control, specifically the husband's authority over the wife. This is in line with other studies which showed that women adhere to traditional gendered roles such as being the primary caregivers, housewives and mothers with men as the key decision-makers in the household (33, 61). As a result, women have low agency in terms of their health-seeking behaviors (46). Indeed, studies in SSA have shown how the familial control exerted by men could impede or facilitate women's access to breast cancer detection services (45, 46). In contrast, some studies have shown that married women are more likely to be more knowledgeable about breast cancer hence detect the disease early (19, 53). Therefore, further studies are required to explore and understand how marital status intersects with gender in breast cancer detection.

From our study, we also found out that gender intersects with social relations. The social relations between a woman and a female health worker who is also resident in the same community and share the same social fabric with the women were highlighted as a major factor shaping women's preference and interaction with the female health worker. Although existing studies identify the use of female healthcare workers to promote breast cancer detection as beneficial (50, 58), from our findings, we identified that the relations between the service users and PHC workers need to be fully understood for sustainability of early detection programs. Another intersecting factor was the link to professionalism within the health workforce. Although recognizing individual preference and autonomy should be embedded within an ideal healthcare system, this choice is not always possible for several reasons. Such reason include the professionalism ascribed to PHCPs and paternalistic communication styles that undermine a woman's agency (24, 62).

### Recommendation- Gendered Approach

Our study agrees with existing literature that health systems, in this case, the Uganda PHC system, is not gender neutral (33). As such, we recommend that the gender disparities perpetuated within the PHC system in Uganda can be effectively addressed by taking a gendered approach. There is increasing recognition of the importance of a gendered approach in policy and practice relating to women's health (63). We posit that a gendered approach for breast cancer detection involves an understanding of how gender is individually and socially constructed and addressing the underlying power relations that perpetuate such gender constructions. Our study findings revealed that the gender constructions of a woman are influenced by several factors, such as the authority wielded by a man over his wife and professionalism ascribed to PHCPs, which undermine women's agency. A gendered approach challenges the existing power hierarchy that positions professional health workers as experts over women's health. Feminist international health movements and platforms such as Beijing Declaration, 1995, challenged the notion that women are passive recipients of care and argued for an active role in shaping women centered care, advocacy and treatment. However, 25 years later there is still much work to do in many countries globally (64). We recommend that to achieve this gendered approach, PHC workers are not only trained on the technical knowledge and skills of breast cancer detection, but their training should be culturally grounded in the reality of the women in the communities in which they serve. In addition to training PHC workers there is also a need to train men in breast cancer detection. The involvement of men in breast cancer detection through sensitization and active involvement as champions of breast cancer detection has been highlighted from past studies (16, 50). Such training and active involvement of men in breast cancer detection have the potential to level the power relations and perpetuate the reality that breast cancer affects women (women-centered) not based on their gendered roles, thus strengthening their agency regarding utilizing breast cancer detection services. Although not framed as a gendered approach, a study in Ghana also identified the need for such woman-centeredness in breast cancer detection (46). Lastly, applying a critical framework like intersectionality is crucial to embed a gendered approach into practice (28, 33). The use of an intersectionality lens in our study enabled us to understand how to address the gender disparities created by the multiple, overlapping social stratifiers and their interconnectedness.

### Strengths and Limitations

The key strength of our study is that, to date, no known study has explicitly focused on addressing the gender disparities attributed to health workers' gender in breast cancer detection in Uganda. This makes the findings of this study not only novel for the Uganda context but has potential for other SSA countries. Also, as studies in HICs have focused on gender disparities due to breast cancer incidence in males vs. females, our findings have the potential to inform a gendered approach for breast cancer detection in HICs, specifically for ethnic-minority populations. Also, our use of a qualitative method was a strength as it enabled us to gather rich and in-depth subjective experiences and perspectives. Although criticized for its subjectivity and inability to produce generalizable data in the same form as the quantitative method (65, 66), the qualitative method recognizes that this subjectivity is contextual and informed by individuals' experiences and voices (65, 67). Despite the value of interviews for our current study, we recommend future studies with services users to employ creative methods like photovoice, which have been found to be instrumental in illuminating gender issues and intersecting factors in research (32, 33).

Our study also had some limitations. The main limitation is that our study findings were based on the perspectives of PHC workers and other stakeholders in PHC delivery, and did not include women, who are the primary service users. The reason for this is that the findings presented are part of a doctoral research on primary health care delivery and breast cancer detection. Therefore, further studies are required to explore the perspectives of women around this challenge of health workers' gender. Also, we focused only on the PHC system, where is there is limited provision of breast cancer detection services. Although the findings are invaluable, there might be differences in higher health care systems where there might be better provision of breast cancer detection services, specifically in relation to the health worker's approachability and professionalism. Also, our study only focused on one town council in Uganda, which may limit the generalizability of the findings, but there is an opportunity for the transferability of our findings (68) to other settings.

## Conclusion

Our study aimed to provide solutions to address gender disparities associated with health workers' gender in breast cancer detection. We adopted an intersectionality lens to understand how gender intersects with other social stratifiers such as religious beliefs, familial control, health worker's approachability, and professionalism within the health workforce. Therefore, due to the complex and dynamic nature of perspectives around the sex of health workers and the context specificity of social constructions, we recommend the need for a gendered-based approach to enhance effective cancer detection and intervention and promote early care-seeking. Taking a gendered approach will ensure that breast cancer detection programs are context-appropriate, cognizant of the prevailing cultural norms, and do not restrict women's access to breast cancer detection services. The findings from this study have great implications for breast cancer control in Uganda and other LMICs and HICs alike. Also, for African communities in HICs, the sociocultural characteristics of the women and the wider community need to be considered in the design and implementation of breast cancer detection or control programs.

## Data Availability Statement

The datasets presented in this article are not readily available because consent provided by study participants did not cover sharing of the dataset with a third-party. Requests to access the datasets should be directed to d.ikhile@bsms.ac.uk.

## Ethics Statement

The studies involving human participants were reviewed and approved by Nottingham Trent University College of Business, Law and Social Sciences Research Ethics Committee. The participants provided their verbal and audio-recorded informed consent to participate in this study.

## Author Contributions

DI conceptualized the study, designed the data collection tool, collected the data, and conducted the analysis with guidance from DM, SS-S, and LG. DI and DO drafted the initial manuscript and the themes were further refined and defined. All authors contributed to subsequent drafts and approved the submitted version.

## Funding

The published data were collected as part of DI's doctoral research supported by the Nottingham Trent University Vice Chancellors scholarship 2016-2019. DI is currently funded by the National Institute for Health Research (NIHR) Applied Research Collaboration Kent, Surrey, Sussex.

## Author Disclaimer

The views expressed are those of the author(s) and not necessarily those of the NHS, the NIHR or the Department of Health and Social Care.

## Conflict of Interest

The authors declare that the research was conducted in the absence of any commercial or financial relationships that could be construed as a potential conflict of interest.

## Publisher's Note

All claims expressed in this article are solely those of the authors and do not necessarily represent those of their affiliated organizations, or those of the publisher, the editors and the reviewers. Any product that may be evaluated in this article, or claim that may be made by its manufacturer, is not guaranteed or endorsed by the publisher.
